# Supplementary material for: Empirical methods for controlling false positives and estimating confidence in ChIP-Seq peaks
Source: BMC Bioinformatics. 2008 Dec 5;9:523. doi: 10.1186/1471-2105-9-523 (PMC2628906; doi:10.1186/1471-2105-9-523)
Supplement: Additional File 1 — A variety of html documents from the USeq web site detailing the available applications, their best usage, output file type descriptions, command line menus, etc. [file 1471-2105-9-523-S1.zip › USeqUserGuides/JavaDoc.zip!/JavaDoc/trans/cel/QuantileNormalization.html]

QuantileNormalization


|  |  |  |
| --- | --- | --- |
| **Overview** \| **Package** \| **Class** \| **Use** \| **Tree** \| **Deprecated** \| **Index** \| **Help** | |  |
| **PREV CLASS**   **NEXT CLASS** | **FRAMES**    **NO FRAMES**     **All Classes** |  |
| SUMMARY: NESTED \| FIELD \| CONSTR \| METHOD | DETAIL: FIELD \| CONSTR \| METHOD |  |


---


## trans.cel Class QuantileNormalization

```
java.lang.Object
  trans.cel.QuantileNormalization
```

---

``` public class QuantileNormalization extends java.lang.Object ```

Performs quantile normalization on arrays of double values. Arrays must be of
equal length.
Note: median scaling can introduce an annoying periodicity into the intensity measurements that is apparent in histograms.
This is not a bug.
An implementation of:
Bolstad BM, Irizarry RA, Astrand M, Speed TP.
A comparison of normalization methods for high density oligonucleotide array data based on variance and bias.
Bioinformatics. 2003 Jan 22;19(2):185-93.

---

**Constructor Summary**

`QuantileNormalization()`

`QuantileNormalization(java.io.File[] files)`


| **Method Summary** | |
| --- | --- |
| `void` | `calculateChromControlSpecificMedian(java.lang.String[] chrNameControls)`             Given chromosome names (e.g. |
| `double` | `calculateMedianOfChromosome(Quantile[] intensities, java.lang.String chromosomeName)`             Looks in the tpmapInfo for the appropriate chromosome, extracts the intensities from the Quantile[], calculates a median. |
| `float[][]` | `extractNormalizedValues()`             Extracts out the normalized values regenerating the original float[][] except the scores have been quantile normalized. |
| `float[]` | `fetchControlIntensities(Quantile[] q)`             Uses the control indexes to fetch the control intensities from the Quantile[]. |
| `static double` | `fetchMedianQuantileValue(Quantile[] m)`             Calculates the median intensity of a sorted Quantile[]. |
| `java.io.File[]` | `getCelpFiles()` |
| `ControlStats[]` | `getControlStats()` |
| `float[][]` | `getIntensities()` |
| `Quantile[][]` | `getQuantiles()`             Fetches an array of sorted normalized Quantiles |
| `float` | `getTargetMedian()` |
| `boolean` | `isUseMMData()` |
| `void` | `loadIntensityArrays(java.io.File[] celaMapFiles)`             Fetch intensity arrays from .celaMap files. |
| `static void` | `main(java.lang.String[] args)` |
| `void` | `makeControlStats()`             Instantiates a ControlStats object for each Quantile[] object. |
| `static Quantile[][]` | `makeQuantiles(float[][] values)`             Makes a Quantile[][] given float[][] where the quantile.position is assigned its array index position and the quantile.value is assigned the float value. |
| `void` | `medianScaleIntensities()`             Median scale current intensity values. |
| `static void` | `normalizeQuantileArrayScores(Quantile[][] quantiles)`             Takes arrays of Quantile, and averages the values for a given array index. |
| `static float` | `percentile(Quantile[] sortedQs, double percentile)`             Returns the value of a given percentile from a SORTED Quantile array. |
| `void` | `prependTargetMedianInControlStats()` |
| `void` | `printIntensityStats()`             Prints statistics about the current intensity float[][] arrays |
| `void` | `quantileNormalize()`             Performs a median scaling then quantile normalization of the loaded float[replicas][intensities]. |
| `void` | `saveAndNullQuantiles()`             Takes the Quantile[][], fetches the float[] values for each, nulls the Quantile[], saves the float[] to disk. |
| `void` | `saveIntensities()`             Saves intensities, one float[] for each replica. |
| `void` | `scaleQuantileArraysByControlIntensities()`             Scales a quantile array that is sorted by original position in the MapFeature[] using the median value of the control intensities. |
| `static void` | `scaleQuantileArraysByMedian(Quantile[][] quantiles, float targetMedian)`             Takes arrays of Quantile that have been sorted by value, and scales their values to the target median. |
| `void` | `setControlChromosomeNames(java.lang.String[] controlChromosomeNames)` |
| `void` | `setControlIndexes(int[][] controlIndexes)` |
| `void` | `setIntensities(float[][] intensities)` |
| `void` | `setModelValuesForScaling(double[] modelValuesForScaling)` |
| `void` | `setQuantiles(Quantile[][] quantiles)` |
| `void` | `setScaleByControlStats(boolean scaleByControlStats)` |
| `void` | `setTargetMedian(float targetMedian)` |
| `void` | `setTpmapInfo(java.util.ArrayList tpmapInfo)` |
| `void` | `setUseMMData(boolean useMMData)` |
| `void` | `sortQuantileArrays(Quantile[][] q)`             Sorts quantile arrays based on comparator, either position or intensity value. |
| `void` | `splineNormalize()`             Uses a cubic spline and user defined regions to non-linearly scale the data. |
| `static float[]` | `transformPMMM(float[] f)`             Performs a max(PM-MM,1) on the float array where MM is just after each PM. |

**Methods inherited from class java.lang.Object**

`equals, getClass, hashCode, notify, notifyAll, toString, wait, wait, wait`

**Constructor Detail**

### QuantileNormalization

```
public QuantileNormalization()
```

---


### QuantileNormalization

```
public QuantileNormalization(java.io.File[] files)
```


**Method Detail**

### main

```
public static void main(java.lang.String[] args)
```

---


### quantileNormalize

```
public void quantileNormalize()
```

:   Performs a median scaling then quantile normalization of the loaded float[replicas][intensities].

---


### splineNormalize

```
public void splineNormalize()
```

:   Uses a cubic spline and user defined regions to non-linearly scale the data.

---


### prependTargetMedianInControlStats

```
public void prependTargetMedianInControlStats()
```

---


### calculateChromControlSpecificMedian

```
public void calculateChromControlSpecificMedian(java.lang.String[] chrNameControls)
```

:   Given chromosome names (e.g. chrGapdh, chrActb) will calculate the median intensities.
    Sets the values and names in the ControlStats[]. Don't forget to call makeControlStats().

---


### makeControlStats

```
public void makeControlStats()
```

:   Instantiates a ControlStats object for each Quantile[] object.

---


### calculateMedianOfChromosome

```
public double calculateMedianOfChromosome(Quantile[] intensities,
                                          java.lang.String chromosomeName)
```

:   Looks in the tpmapInfo for the appropriate chromosome, extracts the intensities from the Quantile[], calculates a median.
    Returns -1 if no chromosome found.

---


### medianScaleIntensities

```
public void medianScaleIntensities()
```

:   Median scale current intensity values.

---


### printIntensityStats

```
public void printIntensityStats()
```

:   Prints statistics about the current intensity float[][] arrays

---


### loadIntensityArrays

```
public void loadIntensityArrays(java.io.File[] celaMapFiles)
```

:   Fetch intensity arrays from .celaMap files.

---


### saveIntensities

```
public void saveIntensities()
```

:   Saves intensities, one float[] for each replica.

---


### transformPMMM

```
public static float[] transformPMMM(float[] f)
```

:   Performs a max(PM-MM,1) on the float array where MM is just after each PM.

---


### saveAndNullQuantiles

```
public void saveAndNullQuantiles()
```

:   Takes the Quantile[][], fetches the float[] values for each, nulls the Quantile[], saves the float[] to disk.
    Transforms the data if mm is present.

---


### makeQuantiles

```
public static Quantile[][] makeQuantiles(float[][] values)
```

:   Makes a Quantile[][] given float[][] where the quantile.position is assigned its
    array index position and the quantile.value is assigned the float value.

---


### normalizeQuantileArrayScores

```
public static void normalizeQuantileArrayScores(Quantile[][] quantiles)
```

:   Takes arrays of Quantile, and averages the values for a given array index.

---


### scaleQuantileArraysByMedian

```
public static void scaleQuantileArraysByMedian(Quantile[][] quantiles,
                                               float targetMedian)
```

:   Takes arrays of Quantile that have been sorted by value, and scales their values to the target median.
    This can introduce an annoying periodicity into the data.

---


### scaleQuantileArraysByControlIntensities

```
public void scaleQuantileArraysByControlIntensities()
```

:   Scales a quantile array that is sorted by original position in the MapFeature[]
    using the median value of the control intensities.
    Be sure the qn array is sorted by position!

---


### fetchControlIntensities

```
public float[] fetchControlIntensities(Quantile[] q)
```

:   Uses the control indexes to fetch the control intensities from the Quantile[].
    Assumes the Quantile[] is sorted by position, not value!
    Takes the median of duplicates.

---


### fetchMedianQuantileValue

```
public static double fetchMedianQuantileValue(Quantile[] m)
```

:   Calculates the median intensity of a sorted Quantile[].

---


### percentile

```
public static float percentile(Quantile[] sortedQs,
                               double percentile)
```

:   Returns the value of a given percentile from a SORTED Quantile array.
    Percentile is from 0-1, ie 0.95 and is according to Lentner, 1982.

---


### sortQuantileArrays

```
public void sortQuantileArrays(Quantile[][] q)
```

:   Sorts quantile arrays based on comparator, either position or intensity value.

---


### extractNormalizedValues

```
public float[][] extractNormalizedValues()
```

:   Extracts out the normalized values regenerating the original float[][] except
    the scores have been quantile normalized.

---


### getQuantiles

```
public Quantile[][] getQuantiles()
```

:   Fetches an array of sorted normalized Quantiles

---


### getIntensities

```
public float[][] getIntensities()
```

---


### setIntensities

```
public void setIntensities(float[][] intensities)
```

---


### getTargetMedian

```
public float getTargetMedian()
```

---


### setTargetMedian

```
public void setTargetMedian(float targetMedian)
```

---


### setQuantiles

```
public void setQuantiles(Quantile[][] quantiles)
```

---


### setControlIndexes

```
public void setControlIndexes(int[][] controlIndexes)
```

---


### isUseMMData

```
public boolean isUseMMData()
```

---


### setUseMMData

```
public void setUseMMData(boolean useMMData)
```

---


### getControlStats

```
public ControlStats[] getControlStats()
```

---


### setTpmapInfo

```
public void setTpmapInfo(java.util.ArrayList tpmapInfo)
```

---


### setScaleByControlStats

```
public void setScaleByControlStats(boolean scaleByControlStats)
```

---


### setControlChromosomeNames

```
public void setControlChromosomeNames(java.lang.String[] controlChromosomeNames)
```

---


### setModelValuesForScaling

```
public void setModelValuesForScaling(double[] modelValuesForScaling)
```

---


### getCelpFiles

```
public java.io.File[] getCelpFiles()
```


---


|  |  |  |
| --- | --- | --- |
| **Overview** \| **Package** \| **Class** \| **Use** \| **Tree** \| **Deprecated** \| **Index** \| **Help** | |  |
| **PREV CLASS**   **NEXT CLASS** | **FRAMES**    **NO FRAMES**     **All Classes** |  |
| SUMMARY: NESTED \| FIELD \| CONSTR \| METHOD | DETAIL: FIELD \| CONSTR \| METHOD |  |


---
